# Characterization of spring and durum wheat using non-destructive synchrotron phase contrast X-ray microtomography during storage

**DOI:** 10.1038/s41538-024-00271-0

**Published:** 2024-05-18

**Authors:** Navanth S. Indore, Chithra Karunakaran, Digvir S. Jayas, Jarvis Stobbs, Miranda Vu, Kaiyang Tu, Omar Marinos

**Affiliations:** 1 Biosystems Engineering, University of Manitoba, Winnipeg, MB Canada; 2 Canadian Light Source Inc., Saskatoon, SK Canada; 3 President’s Office, A762 University Hall, University of Lethbridge, Lethbridge, AB Canada

**Keywords:** Engineering, Materials science

## Abstract

Post-harvest losses during cereal grain storage are a big concern in both developing and developed countries, where spring and durum wheat are staple food grains. Varieties under these classes behave differently under storage, which affects their end storage life. High resolution imaging data of dry as well as spoiled seed are not available for any class of wheat; therefore, an attempt was made to generate 3D data for better understanding of seed structure and changes due to spoilage. Six wheat varieties (3 varieties for each class of wheat) were stored for 5 week at 17% moisture content (wb) before scanning. Seeds were also stored in a freezer (-18 °C) for further scanning to determine if any changes occur in the structure of seeds due to freezing. Spring varieties of wheat performed better than durum varieties and freezing did not affect seed structure. Data could also help plant breeders to develop varieties that do not easily spoil, adjust grain processing techniques, and develop post-harvest recommendations for other wheat varieties.

## Introduction

The world population is expected to grow up to 9.1 billion by 2050, hence more food is required to feed the growing population. As per FAO, the demand for food (cereals and animal feed) is expected to reach 3 billion tonnes in 2050; therefore, overall food production needs to increase by 70%^1^. Cereals are the main staple food for developing and developed nations. This implies significant increases in the annual cereal production; for instance, it would have to grow by almost one billion tonnes. The soaring food demand may exert more pressure on currently depleting natural resources and will be challenging to achieve food security, but prevention or reduction of post harvest losses could be a savior in meeting the food demand, as saved food is equal to food produced. The post-harvest losses are not only limited to physical loss but also quality loss due to fungi (mycotoxins). Presently, post harvest losses in cereals occur ~10–20% in developing and 1–2% in developed countries in storage^2^ and it can range from 1% in well-managed systems to 50% in poorly managed systems^3^. The maximum fraction of all post-harvest losses for cereals in developing countries is in storage losses, which negatively affect farmers’ livelihoods^4^. The high moisture content leads to the growth of fungi on food grains under favorable temperatures for fungi. Changes in the quality of wheat during storage due to biotic and abiotic factors have been reported in studies^5–11^. As per a World Bank report, food grains worth about USD 4 billion are lost every year due to post harvest loss^4^. The storage losses are considered most critical in developing countries^4^.

Canada produced 33.8 million tonnes (Mt) of wheat in 2022, whereas the production of durum and spring wheat was 6.11 and 19.38 Mt^12^, respectively. There is a demand for good quality wheat, and Canada exported ~60.6% of its produced wheat in 2022. Considering post harvest loss of 2% as a developed nation (scientific storage), it would be a loss of 331 million CAD in monetary terms (@490 CAD/t). Post-harvest losses in wheat that occur during storage are two types: quantitative and qualitative. Quantitative loss means loss in dry mass, damage due to insects and fungi, and qualitative loss means loss of nutrition, loss of germination, and infection due to mycotoxins. Wheat is stored after harvesting before consumption. During storage and along the supply chain, moisture content plays an important role in determining its storage life. If the moisture content of grains is higher than the recommended safe storage value, then stored grains are vulnerable to spoilage due to biotic (insect, fungi) and abiotic (temperature, moisture) factors. Wheat grain deterioration is primarily driven by aerobic respiration of fungi because fungi thrive on carbohydrates in the kernel, giving off carbon dioxide (CO_2_), moisture (H_2_O), and heat. High moisture wheat has higher respiration heat than dry wheat and that leads to hot spots and zones of infection. Several studies have been carried out at the macroscale to minimize storage losses in wheat. As seed structure also plays a major role in its defense mechanism against spoilage due to unfavorable conditions in storage, it is essential for microscale understanding of wheat seed structures. For instance, existing kernel structure before storage has a major role to play; if the seeds are already cracked or damaged during handling operations, it leads to entry of moisture and insects^13,14^. Therefore, the study of seed structure becomes an important aspect of grain storage, which is the scope of this study.

Wheat storage is facing enormous challenges^14^; therefore, numerous studies have been undertaken on macro and micro levels to understand the quality and loss assessment of wheat in grain storage across the world^3,11,13,15–20^. The main scope of these studies is to determine safe storage limits, reduce post harvest loss, assess quality during storage, and develop post harvest storage guidelines suited to local conditions. The microlevel studies or measures include the effect of scientific storage (controlled storage, hermetic) on wheat quality and the interaction of abiotic (moisture, temperature, and humidity) and biotic (insects and fungi) factors on grain quality and post harvest losses^21^. These large-scale storage studies require bigger setups and infrastructure for simulating conditions, unlike the microscopic approach. The microscopic method involves destructive and non-destructive approaches, such as the use of X-rays, hyperspectral imaging, and thermal imaging to quantify defects before loading, during storage, or after unloading. These studies have created useful results in the identification of specific fungi, and macrolevel studies mainly focused on large-scale storage systems where the interaction of biotic and abiotic factors was studied on the post storage quality of food grains^22–26^. Besides these developments in quality evaluation of wheat using microscopic methods, there are limitations in exploring required information from the grain samples due to limitations of the system such as sample preparation, low contrast in imaging, time consumption, low resolution, more scanning time, and non-selective wavelength^27,28^. The conventional X-ray imaging system^23,29–32^ used in characterization of various structural features of wheat was also reported. In wheat seed, crease depth and morphology were found to be responsible for diseases such as black spots that infect grains particularly at the crease^33^. Therefore, the use of high-resolution imaging such as synchrotron X-ray imaging has found promising applications in post harvest storage studies. Applications of synchrotron X-ray imaging were found in understanding seed structure, soil science, and plant systems in agriculture, which were reviewed^27^.

To date, there are no high-resolution data available on wheat grain structure that can be used in planning post harvest management strategies, and the present work fills this knowledge gap, where synchrotron phase contrast imaging was used in a post harvest storage study of wheat. The selection of phase contrast imaging was made over X-ray absorption due to its edge enhancement characteristics of low-density materials, because similar applications were found in the characterization of soft tissues, water/nutrient movements and multiphase^27,34–36^. It is difficult to differentiate between spoilage, moisture, and air in absorption X-ray imaging; therefore, phase contrast imaging has been used due to its edge enhancement characteristics. Grain moisture is a crucial parameter in the post harvest storage of wheat, which can be controlled during all unit operations performed before storage. The objective of this study was to gather structural features information from two popular wheat classes (spring and durum) using phase contrast imaging for the characterization of dry and fungal infected seeds. Another objective was to develop a methodology for testing cereal grains using synchrotron imaging in post-harvest grain storage studies, which can be used for other wheat classes and cereal grains. The results of the study could also help plant breeders to develop varieties that do not spoil easily, adjust grain processing techniques such as milling, and develop post harvest recommendations for other wheat varieties.

## Results

The physical condition of moist samples of all six varieties stored for 5 week was assessed by visual observation. Both durum wheat and spring wheat showed varying levels of deterioration during the visual inspection compared to control samples. Durum wheat showed more deterioration in comparison to spring wheat during the visual inspection of wheat kernels. The fungus was visible on some of the germ parts of the AAC Spitfire variety, followed by CDC Defy and AAC Stronghold. In case of spring wheat, AAC Starbuck and AAC Brandon were affected more during storage, but many of the kernels were found to be sound compared to durum wheat, where almost both replicates of samples of AAC Spitfire and CDC Defy showed deterioration. The Faller variety did not show major visible deterioration on the surface of grain kernels.

**Fig. 1:**
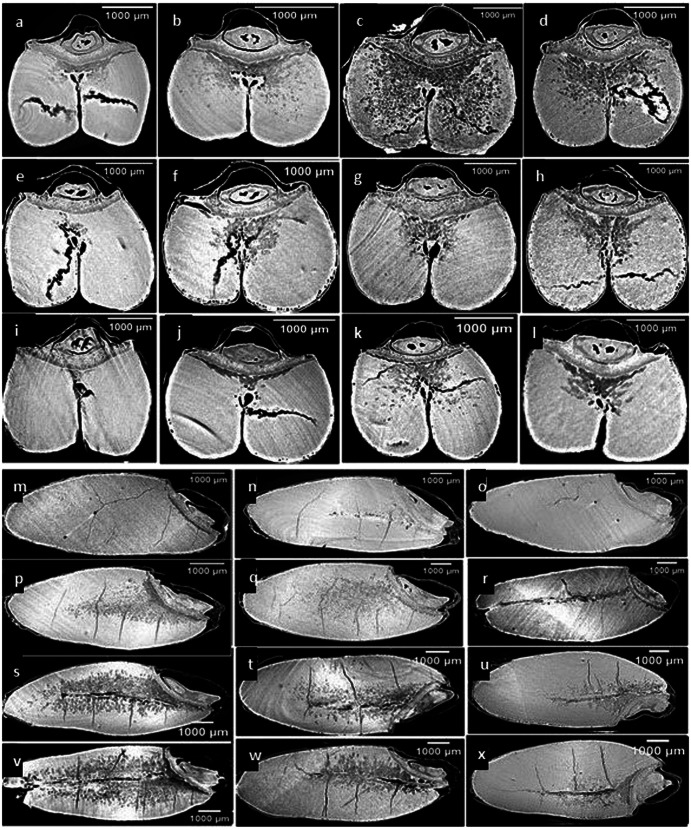
Durum wheat SR-µCT data. AAC Spitfire (**a**–**d**, **m**, **p**, **s**, **v**), CDC Defy (**e**–**h**, **n**, **q**, **t**, **w**), and AAC Stronghold (**i**–**l**, **o**, **r**, **u**, **x**). The storage conditions: control (**a**, **e**, **i**, **m**–**o**), 3 weeks (**b**, **f**, **j**, **p**–**r**), 5 weeks (**c**, **g**, **k**, **s**–**u**), and frozen 5 weeks (**d**, **h**, **l**, **v**–**x**).

**Fig. 2:**
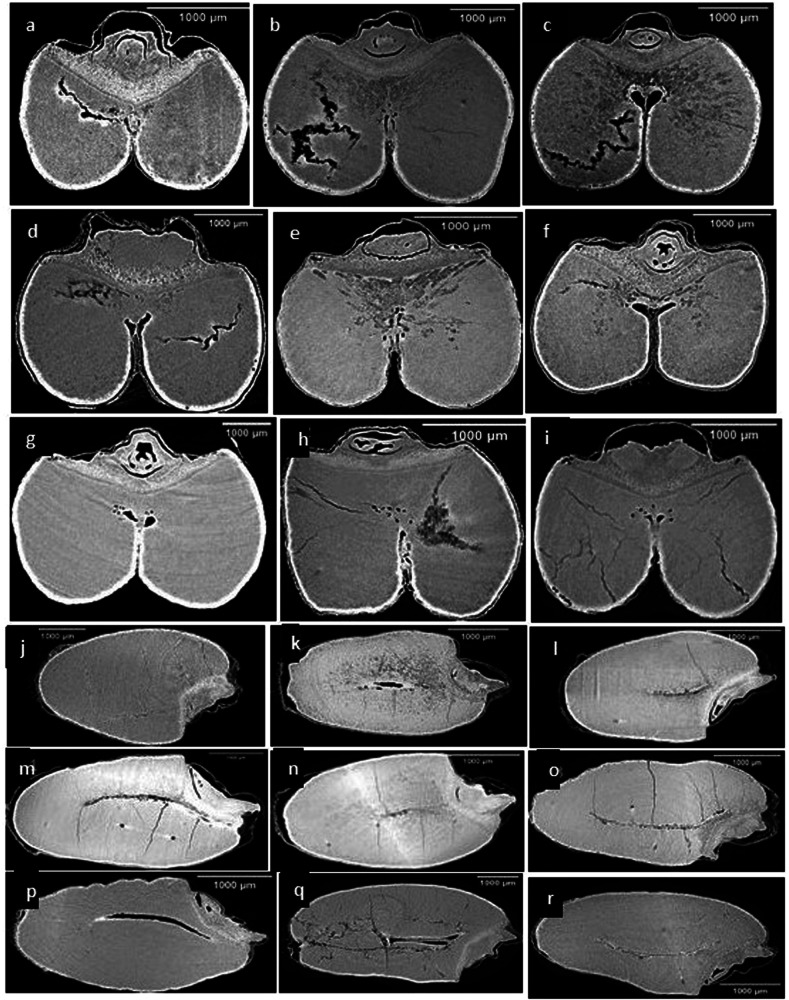
Spring wheat SR-µCT data. AAC Brandon (**a**–**c**, **j**, **m**, **p**), AAC Starbuck (**d**–**f**, **k**, **n**, **q**), and Faller (**g**–**i**, **l**, **o**, **r**). The storage conditions: control (**a**, **e**, **i**, **m**, **n**, **p**), 5 weeks (**b**, **e**, **h**, **k**, **n**, **q**), and frozen 5 weeks (**c**, **f**, **i**, **l**, **o**, **r**).

**Fig. 3:**
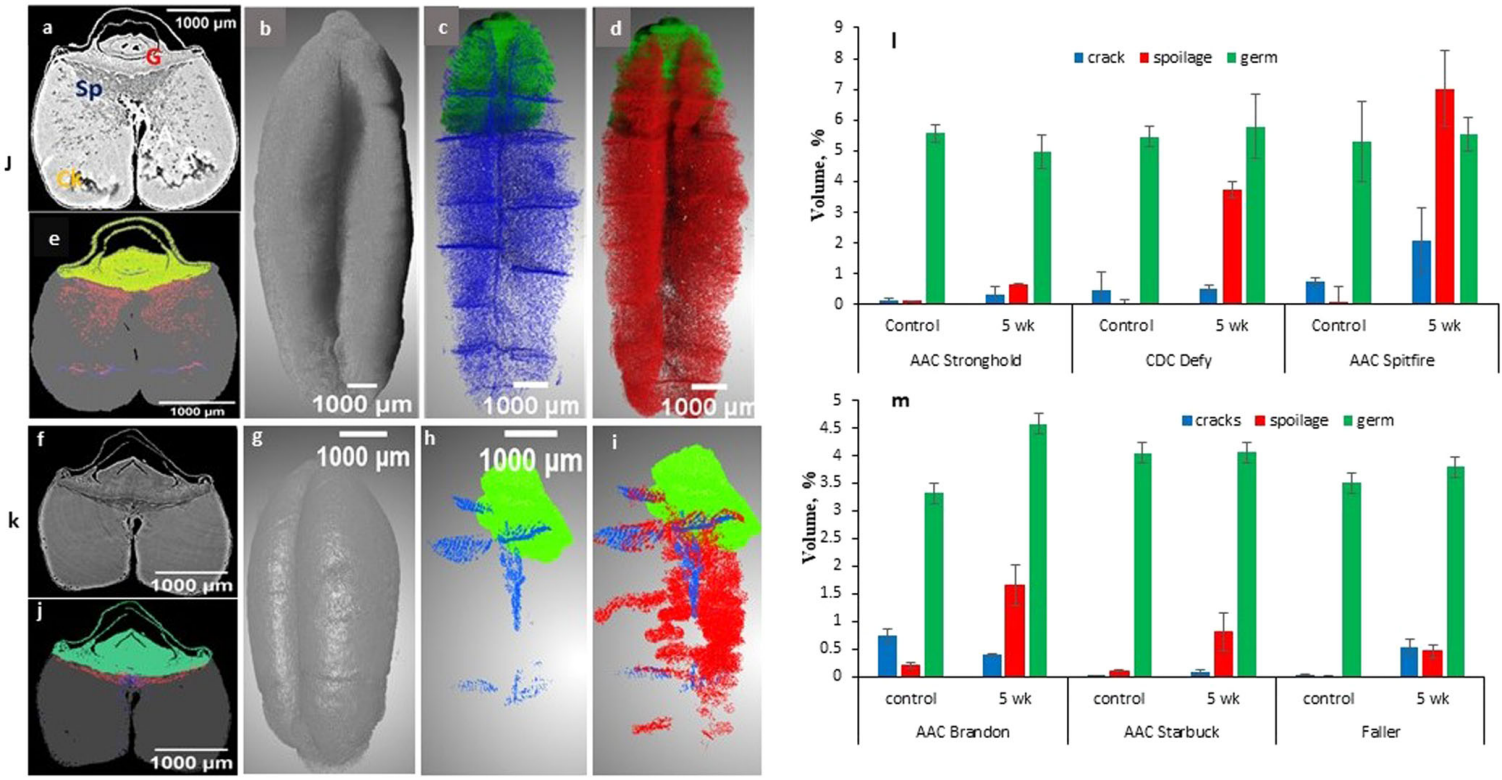
Segmented seed features and measurements of 5-week stored Durum wheat (AAC Spitfire) and Spring wheat (AAC Brandon). **j** AAC Spitfire (**a**: X-ray image and **e**: labeled image), volume rendered image of labeled voxels (**b**: seed, **c**: germ in green, cracks along with pore in blue, and **d**: change due to spoilage in red). **k** AAC Brandon (**f**: X-ray image and **j**: labeled image), volume rendered of labeled voxels (**g**: seed, **h**: germ, cracks along with pores and **i**: change due to spoilage). At the bottom, measured segmented volumes of components for durum wheat (**l**) and spring wheat (**m**). The error bars refer to standard deviation of mean (*n* = 3).

**Fig. 4:**
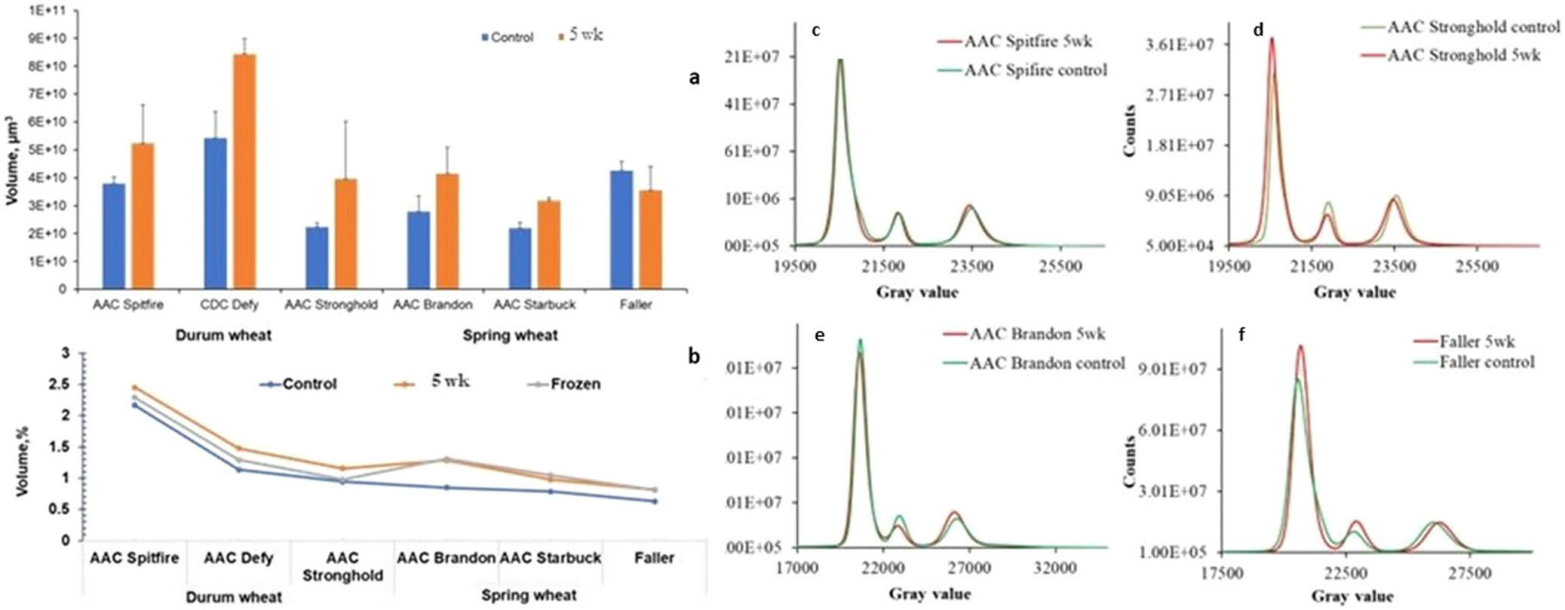
Seed volume, porosity measurements, and histogram comparison between Durum and Spring wheat varieties. Analyzed seed volumes **a** of Durum wheat and spring wheat and porosity **b** of control, 5-week and 5-week frozen (*n* = 20). The error bars refer to the standard deviation of the mean. The histogram of 5-week stored wheat and control (**c**: AAC Spitfire, **d**: AAC Stronghold, **e**: AAC Brandon, **f**: Faller).

**Fig. 5:**
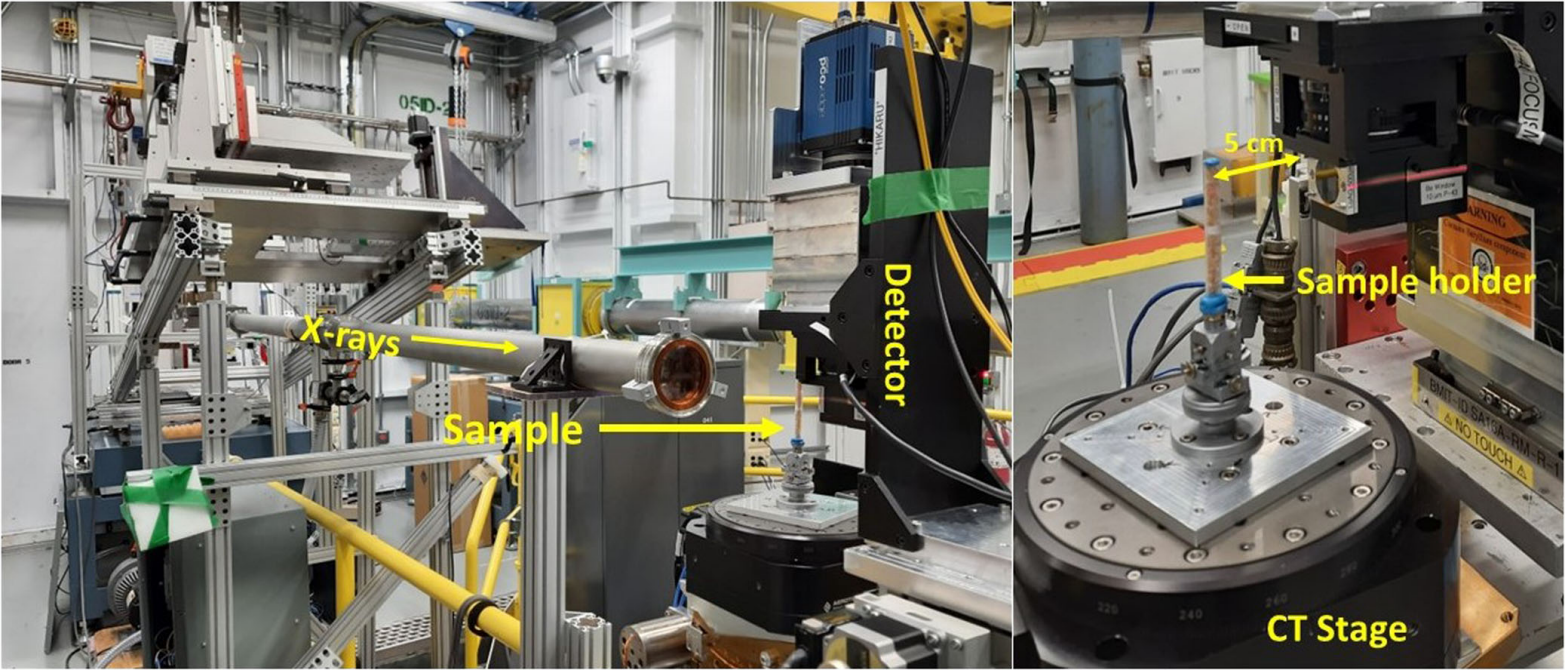
Setup of SR-μCT experiment. Left: SR-µCT beamline and sample setup, and right: enlarged view showing sample and detector.

The scanned seeds using synchrotron X-Ray phase contrast microcomputed tomography (SR-µCT) have shown promising results and revealed important microstructural information. The high-resolution data of both wheat classes were visualized in the form of two types of cross sections, as shown in Figs. 1 and 2, and both durum and spring wheat showed distinctive features in seed structure, size, and shape. The presence of cracks was predominant in all three control samples of durum varieties Fig. 1a, e, i, m, n, o compared to control samples of spring wheat varieties Fig. 2a, d, g. The seed endosperm was found to be more damaged in the AAC Spitfire and CDC Defy than in the AAC Stronghold. In the case of spring wheat, damage or presence of cracks were found but less in comparison to durum wheat. The varieties AAC Starbuck and AAC Brandon had higher anomalies than the Faller variety. Control samples of Faller wheat variety were sounder than the other five varieties of wheat. The seed coat (with aleurone layer) of durum wheat was more affected than spring wheat as storage time increased from 1 to 5 week. The higher deterioration due to fungal infection was clearly visible in durum varieties from 3 week Fig. 1b, f, j, p, q, r to 5 week Fig. 1c, g, k, s, t, u and in frozen samples Fig. 1d, h, l, v, w, x. Also, deterioration was observed in spring varieties from 5 week Fig. 2b, e, h, m, n, o only and changes remain unaltered in frozen samples Fig. 2c, f, i, p, q, r. AAC Spitfire and CDC Defy in durum wheat severely spoiled than the AAC stronghold, and we were able to characterize them based on these differences (Fig. 1). Similarly, in spring wheat, AAC Brandon and AAC Starbucks deteriorated more than Faller with an increase in storage time (Fig. 2). The changes in microstructure might have caused due to spoilage due to fungi, which is visible across damaged seed endosperm in Figs. 1 and 2. The fungi *Aspergillus flavus, A. niger, Circinella umbellata, Gliocladium sp., Penicillium frequentans, P. islandicum*, and *Ulocladium atrum* are found in stored wheat in bulk storage^14^.

The changes in 5-week frozen samples of both wheat classes were accessed by visualizing all six projections Figs. 1d, h, l, v, w, x and 2c, f, i, p, q, r and there was no further deterioration and our hypothesis hold true that freezing of spoiled samples does not alter structure further. These results also validate the varying spoilage among the varieties of the same class. In this study, we mapped changes due to deterioration using high-resolution imaging during post harvest storage in our study. The changes due to spoilage among all varieties were segmented, visualized, and measured as presented in Fig. 3. The 3D visualization of damaged areas due to spoilage of two varieties (AAC Spitfire and AAC Brandon), which have shown more deterioration from each class, is presented in Fig. 3j, k. The volume of segmented features was measured for both wheat classes and is shown in Fig. 3l, m. All three varieties of durum wheat showed higher volume of segmented deterioration compared to spring varieties Fig. 3l. The AAC Spitfire variety had a maximum segmented volume of change due to spoilage followed by CDC Defy and AAC Stronghold. It was also observed that, durum wheat had a comparatively higher germ volume than spring varieties. Most of the deterioration was initiated near the germ and the crease part from early weeks and then spread further. The germ area was the first to be infected with fungal infection and durum had it more than spring which can be one of the reasons of faster spoilage of durum than spring varieties. These findings of measured spoilage volume can be linked to the findings of Fig. 4a, where large variation in seed volume was in durum wheat than spring wheat. CDC Defy had the highest seed volume among all varieties studied whereas, AAC Starbuck had the lowest seed volume. The measured values of air gaps and cracks in seeds are presented in Fig. 4b, where AAC Spitfire had the maximum percentage of air space and Faller had the lowest percentage among all, and the same was verified earlier from 2D projections of both wheat (Fig. 1 and Fig. 2). Increase in airspace values along with change in seed volume from control samples to 5-week stored samples could be an indication of spoilage. The measured airspace values of the frozen samples were almost similar to those of the 5-week stored samples and did not show major variation as per Fig. 4b.

The wheat germ volume was segmented and calculated in our study as shown in Fig. 3l, m. The histogram of 5-week stored and control wheat samples were produced and presented in Fig. 4c–f, and the purpose was to check shifts in the peaks of selected varieties that reflect the deterioration. The first peak in histogram refers to air surrounding and inside the samples, second peak corresponds to sample holder and third peak is the endosperm. The changes due to deterioration lie near or left side of the third peak as referred in previous literature^37^. The maximum shift was observed in the AAC Spitfire as shown in Fig. 4c and minimum in AAC Stronghold (e). The minimum shifts were observed in spring wheat Fig. 4e, f. The shift of histogram in durum wheat was observed, which is a sign of infection due to fungi, where the most affected variety AAC Spitfire showed more shift with respect to the control sample. A similar shift in histogram was first reported for maize seeds in a study^38^.

## Discussion

All agriculture food grains are inherently variable and cannot have the same size and shape and even internal features, as is obvious from the difference in germ volumes in control samples as well as 5-week stored samples. The cracks evolved from the crease of the kernel and propagated across the endosperm and even in some cases breached the seed coat, which could be visible from both longitudinal and cross-sections of both datasets. These uneven imperfections (cracks, holes) in grain kernel might have made them vulnerable to spoilage during storage and the reasons behind this internal damage could be uneven drying, weathering of crop before harvesting, and grain handling operations^14^. As the storage period progressed from 1 week to 5 week, an increase in the level of deterioration was found in all cross sections. The change in the structure of kernel due to spoilage could be visualized by differences in the gray values in the cross-section near the germ, crease, and around cracks.

One change found common in both wheat classes during storage is that deterioration initiated at the crease part of the wheat and then followed the path of the damaged area. Therefore, the crease features (depth, opening) of wheat could have a significant impact on the wheat response to spoilage in storage. It was earlier reported that, wheat seeds affected due to diseases at the crease^29^ and the same was verified from our images of both spring and durum wheat in this study. It is evident from the images that moisture could enter from the physically damaged seed surface and across the crease rather than the seed coat, which was the main cause found in the deterioration of wheat. These observations can be verified from the projections because the best performing spring variety Faller had a tight closed crease along with fewer imperfections in the endosperm.

Image segmentation also revealed that in both classes of wheat, fungal infection starts at the germ area near the crease and spreads or follows the cracks or damaged areas. This evidently suggests that the germ part of wheat is vulnerable to spoilage during storage of wheat and hence loss of viability of seed. This characterization of varieties based on changes in internal components provides critical information about wheat varieties, indicating that each variety in a class can behave differently in grain storage. The deterioration differed in all six varieties but strongly correlated to the existing condition of control samples. SR-µCT is best to differentiate between phases that have low X-rays such as soft tissue, and in this study, we were able to differentiate and segment change due to spoilage and airspace, which have almost similar X-ray absorption.

Recommendation could be drawn from the study that prior understanding of dry samples using synchrotron X-rays is required before long-term storage. High-resolution data of wheat seed would be helpful in the modeling and planning of long-term storage studies. The post-harvest losses could be high if inferior or damaged seeds were stored or selected for long-term grain storage. It is evident from the image analysis of selected varieties that varieties of the same class behave differently. Earlier there was no evidence of how much deterioration in wheat seed structure could happen during post harvest spoilage at the microscopic level, but our study has shown how much actual level of deterioration could happen in wheat if stored at unsafe moisture. The results will have a greater impact on wheat storage because if healthy grain will go into grain storage and storage losses could be reduced significantly. Similar results were reported in durum wheat storage experiment^9^.

Earlier studies of X-ray absorption were unable to differentiate changes due to spoilage inside the wheat, but in this study, it was possible due to phase contrast imaging where phase of low density difference can be visualized^28,35^. It was earlier reported that porosity of dry cereal kernel could not be measured in lab due to inaccessibility of air space^39^, but in this study it was measured in both classes. Conducted research has wide application in the area of grain storage handling where developed methodology in this study would be very useful in testing of storage study samples. Researchers should be able to freeze their samples during long-term storage studies and transport the frozen samples for imaging during the block of time assigned to that study at a synchrotron facility. One more application area is that, collected high-resolution data could be useful in the development of new/improved varieties through breeding programs that have better crease features and less brittle endosperm. These data will help post-harvest engineers develop efficient modeling strategies for better storage for wheat and can be correlated with nutrient changes in wheat. The future scope of this work could be the use of machine learning algorithms in extracting information about the opening of crease and crease depth in different classes of Canadian wheat.

## Methods

### Wheat

In this study, we procured and used commercially available certified seeds of Canada Western Amber Durum wheat (varieties: CDC Defy, AAC Stronghold, and AAC Spitfire) and Canada Western Spring Wheat (varieties: AAC Starbuck, AAC Brandon and Faller) from a company (SeCan, Niverville, MB).

### Storage experiment

Five-hundred-gram samples of each variety were conditioned to a moisture content (mc) of 17% wet basis (wb) by adding distilled water in triplicates and then stored in airtight glass jars (volume 1 L), and these procedures were repeated every week up to f5 week. Then glass jars were stored at ambient conditions where storage temperatures were in the range of 22-25 °C. The mc was determined 24 h after conditioning by oven drying of 10 g samples at 130 °C for 19 h and the samples were reconditioned, if necessary, to maintain at 17 ± 0.5%^40^.

### Synchrotron X-Ray phase contrast microcomputed tomography

#### Sample preparation

No specific sample preparation is required for this imaging technique, where samples such as agricultural food grains can be scanned noninvasively. Fifteen to twenty grain kernels of each variety were randomly selected from two replicate jars and placed inside a plastic tube. The grains of the same variety were placed inside the tube with the bottom having replicate 2 and the top having replicate 1 for the identification of acquired image slices (slice number 1 (top) - slice number 13 (bottom). The tube ends were closed by a reusable adhesive to prevent the dehydration of the seeds during scanning and then placed on the sample stage. The sample was eccentrically aligned perfectly on the stage to keep it in the field of view of the beam, and then the sample was mounted on a rotating stage between the detector and X-ray beam. The rotating stage was used to change the orientation of the sample in 0.06° steps with respect to the incident white beam of 20 keV. The distance between the sample and detector is important in phase contrast imaging for better contrast due to edge enhancement, and the distance was 5 cm (Fig. 5). The detailed principles and theory of X-ray phase contrast imaging can be found elsewhere^41–43^.

### Data acquisition and image processing

The SR-µCT imaging of the samples was carried out using a BMIT-BM beamline at the Canadian Light Source (CLS), Saskatoon, Canada. Two filters Al (0.800-mm thick) and Md (0.076 mm) were used before the monochromator to obtain a filtered white beam of energy of about 20.0 keV. The transmitted X-ray images were converted into visible images by a combination of a scintillator (LuAg 500) and a detector (PCO Edge 5.0, AA-40) for 3.6 µm resolution in the study (Fig. 3). The collected projection images were corrected for the dark signal from the detector (without X-ray beam) and the flat signal (with X-ray beam and no sample) to account for the imperfections in the beam and inhomogeneity in the scintillator screens. The entire sample was made of 13 slices with each slice consisting of 3000 images. The images of flat and dark signals were recorded before and after recording the CT images and the average of the flat and dark images was used for normalization^28,35,44^. Phase retrieval was performed from a single phase-contrast image at each projection angle using Paganin’s method using the UFO-kit software (Tofu 0.12, Karlsruhe Institute of Technology, Karlsruhe, Germany). Vertical stitching of all 13 slices was carried out after the reconstruction of data using the same software, where all projections were stitched vertically by finding overlap by matching two consecutive data sets using the ez_helper module of the UFO- kit. As the dataset was large and data were binned 4 × 4 to size <4 Gb using Fiji (2.9.0)^45^.

**Fig. 6:**
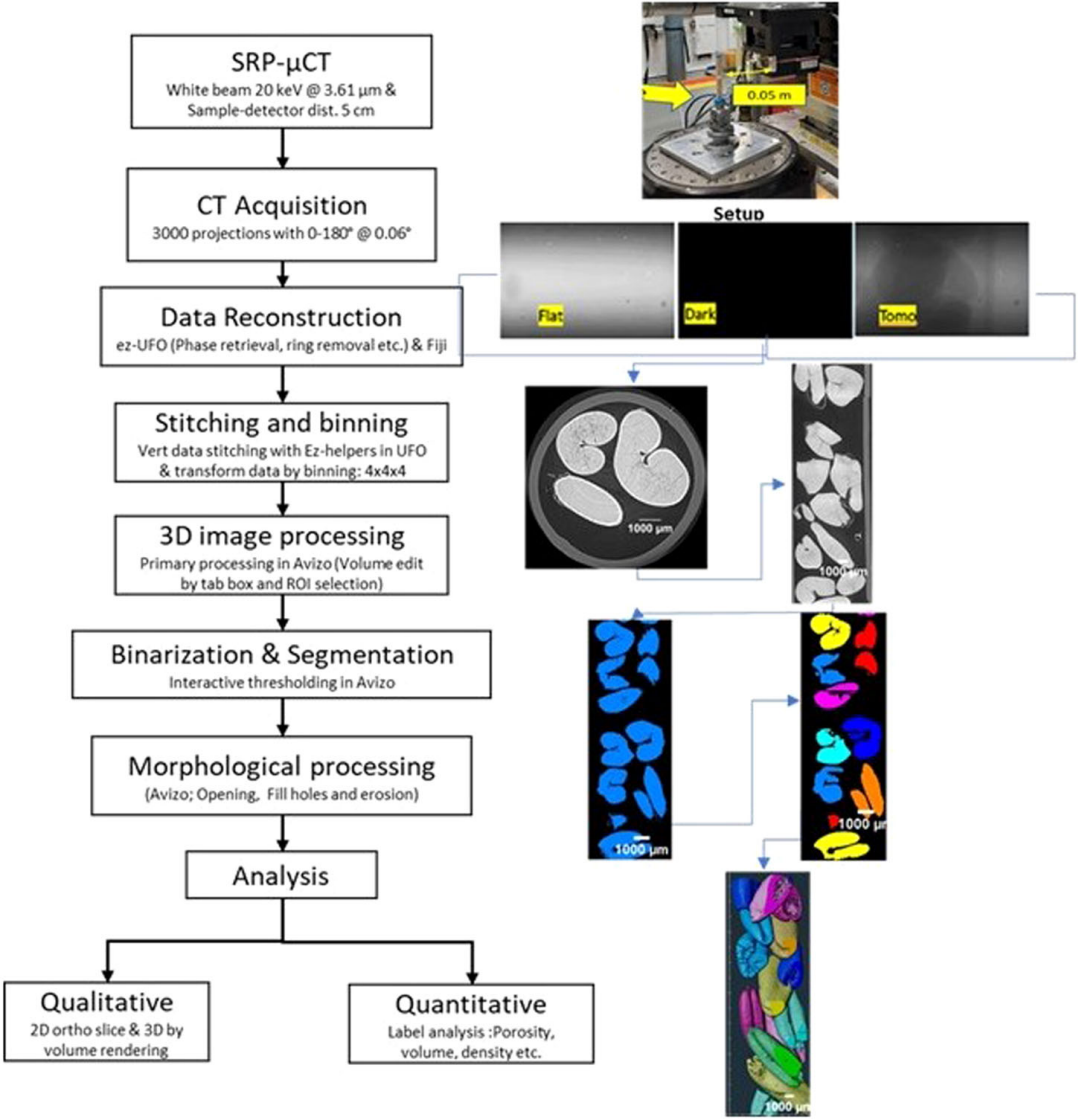
Developed image processing procedure of SR-μCT data. Left: developed image processing pipeline of SR-µCT data using Avizo and right: visualization of Avizo image processing steps.

Binarization using interactive thresholding was performed for each data set to segment seeds from the air space for further analysis. The thresholded image was then used for morphological operations (opening, closing, and fill holes) to remove unwanted pixels from seed boundaries, which is a prerequisite for performing seed label analysis. The label analysis of Avizo improved the thresholded images that were used to determine: seed volume, size dimensions, and porosity (Fig. 6). The porosity of the seed samples was determined using the volume fraction module of Avizo by analyzing the thresholded seed images. Similar image processing was performed in past studies^28,35,46^. Individual seed segmentation was achieved using a customized ROI tool of the ORS Dragonfly (2021.3, Object Research Systems, Montreal, QC) module by selecting a target pixel. Before segmentation, the three most spoiled seeds of each variety were selected as replication by visualizing the collected projection. Three control seeds were randomly selected for segmentation of each variety. In the first step, separate ROIs were created for each component of interest (germ, air space, change in structure/spoilage, and whole seed) in the segmentation editor of the ORS dragonfly. The defined range (global thresholding) based on histogram intensity was used for painting or selection of component pixels, as shown in Fig. 6. Labeled pixels were measured by an analysis module for the determination of the volume of each component of the seed.

### Reporting summary

Further information on research design is available in the Nature Research Reporting Summary linked to this article.

### Supplementary information

Reporting Summary

## Data Availability

High-resolution imaging data are available on request from the corresponding author.

## References

[1] FAO. 2050: A third more mouths to feed. *Food and Agriculture Organization of the United Nations* (2009).

[2] Hodges RJ, Buzby JC, Bennett B (2011). Postharvest losses and waste in developed and less developed countries: Opportunities to improve resource use. J. Agric. Sci..

[3] Jayas DS (2012). Storing grains for food security and sustainability. Agric. Res..

[4] Kumar D, Kalita P (2017). Reducing postharvest losses during storage of grain crops to strengthen food security in developing countries. Foods.

[5] Worku AF, Kalsa KK, Abera M, Habtu NG (2019). Effects of storage strategies on physicochemical properties of stored wheat in Ethiopia. AIMS Agric. Food.

[6] Yuan Q-S (2018). Variation in the microbiome, trichothecenes, and aflatoxins in stored wheat grains in Wuhan, China. Toxins (Basel).

[7] Zhang S-B (2017). Physiochemical changes in wheat of different hardnesses during storage. J. Stored Prod. Res..

[8] Schmidt M (2016). Impact of fungal contamination of wheat on grain quality criteria. J. Cereal Sci..

[9] Nithya U, Chelladurai V, Jayas DS, White NDG (2011). Safe storage guidelines for durum wheat. J. Stored Prod. Res..

[10] Schroth E, Muir WE, Jayas DS, White NDG, Abramson D (1998). Storage limit of wheat at 17% moisture content. Can. Agric. Eng..

[11] Wallace HAH, Sholberg PL, Sinha RN, Muir WE (1983). Biological, physical and chemical changes in stored wheat. Mycopathologia.

[12] Statistics Canada. *Model-based Principal Field Crop Estimates*. https://www150.statcan.gc.ca/n1/daily-quotidien/220914/dq220914b-eng.htm (2022).

[13] Neme K, Mohammed A (2017). Mycotoxin occurrence in grains and the role of postharvest management as a mitigation strategies. A review. Food Control.

[14] Moustafa, S. M. N., Alhaithloul, H. A. S. & Abdelzaher, H. M. A. *Global Wheat Production*10.5772/intechopen.75585 (2018).

[15] Cheyed SH (2020). Effect of storage method and period on vitality and vigour of wheat seed. Indian J. Ecol..

[16] Kibar H (2015). Influence of storage conditions on the quality properties of wheat varieties. J. Stored Prod. Res..

[17] Tian PP, Lv YY, Yuan WJ, Zhang SB, Hu YS (2019). Effect of artificial aging on wheat quality deterioration during storage. J. Stored Prod. Res..

[18] Karunakaran C, Muir WE, Jayas DS, White NDG, Abramson D (2001). Safe storage time of high moisture wheat. J. Stored Prod. Res..

[19] Moreno-Martínez E, Rivera A, Badillo MV (1998). Effect of fungi and fungicides on the preservation of wheat seed stored with high and low moisture content. J. Stored Prod. Res..

[20] Vimala Bharathi SK, Vishnu Priya V, Eswaran V, Moses JA, Sujeetha ARP (2017). Insect infestation and losses in stored food grains. Ecol., Environ. Conserv..

[21] Jian F, Jayas DS (2012). The ecosystem approach to grain storage. Agric. Res..

[22] Jayas, D. S., Paliwal, J., Erkinbaev, C., Ghosh, P. K. & Karunakaran, C. *Computer Vision Technology for Food Quality Evaluation.* p. 385–412 (Elsevier, 2016).

[23] Neethirajan S, Karunakaran C, Symons S, Jayas DS (2006). Classification of vitreousness in durum wheat using soft X-rays and transmitted light images. Comput. Electron Agric..

[24] Singh CB, Jayas DS, Paliwal J, White NDG (2012). Fungal damage detection in wheat using short-wave near-infrared hyperspectral and digital colour imaging. Int J. Food Prop..

[25] Bechtel DB, Zayas I, Dempster R, Wilson JD (1993). Size distribution of starch granules isolated from Hard Red Winter and Soft Red Winter wheats. Cereal Chem..

[26] Narvankar DS, Singh CB, Jayas DS, White NDG (2009). Assessment of soft X-ray imaging for detection of fungal infection in wheat. Biosyst. Eng..

[27] Indore NS, Karunakaran C, Jayas DS (2022). Synchrotron tomography applications in agriculture and food sciences research: a review. Plant Methods.

[28] Karunakaran C (2015). Factors influencing real time internal structural visualization and dynamic process monitoring in plants using synchrotron-based phase contrast X-ray imaging. Sci. Rep..

[29] Le, T. D. Q., Alvarado, C., Girousse, C., Legland, D. & Chateigner-Boutin, A. L. Use of X-ray micro computed tomography imaging to analyze the morphology of wheat grain through its development. *Plant Methods* **15**, 84 (2019). 10.1186/s13007-019-0468-y PMC6668075 31384289

[30] Hughes, N. et al. Non-destructive, high-content analysis of wheat grain traits using X-ray micro computed tomography. *Plant Methods* **13**, 76 (2017). 10.1186/s13007-017-0229-8 PMC5664813 29118820

[31] Neethirajan S, Jayas DS, White NDG, Zhang H (2008). Investigation of 3D geometry of bulk wheat and pea pores using X-ray computed tomography images. Comput. Electron. Agric..

[32] Karunakaran C, Jayas D, White N (2003). Soft X-ray image analysis to detect wheat kernels damaged by Plodia interpunctella (Lepidoptera: Pyralidae). Sci. Aliments.

[33] Radchuk V, Weier D, Radchuk R, Weschke W, Weber H (2011). Development of maternal seed tissue in barley is mediated by regulated cell expansion and cell disintegration and coordinated with endosperm growth. J. Exp. Bot..

[34] Lee SJ, Kim Y (2008). In vivo visualization of the water-refilling process in xylem vessels using x-ray micro-imaging. Ann. Bot..

[35] Brar GS (2019). Showcasing the application of synchrotron-based X-ray computed tomography in host-pathogen interactions: The role of wheat rachilla and rachis nodes in Type-II resistance to Fusarium graminearum. Plant Cell Environ..

[36] Lahlali R (2015). Synchrotron based phase contrast X-ray imaging combined with FTIR spectroscopy reveals structural and biomolecular differences in spikelets play a significant role in resistance to Fusarium in wheat. BMC Plant Biol..

[37] Indore NS, Chaudhry M, Jayas DS, Paliwal J, Karunakaran C (2024). Non-destructive assessment of microstructural changes in Kabuli chickpeas during storage. Foods.

[38] Rawson SD, Maksimcuka J, Withers PJ, Cartmell SH (2020). X-ray computed tomography in life sciences. BMC Biol.

[39] Chang C (1987). Measuring density and porosity of grain kernel using gas pycnometer. Cereal Chem..

[40] ANSI/ASABE. Moisture Measurement — Unground Grain and Seeds. *ASABE Standards* 2–4 (2017).

[41] Willmott, P. *An Introduction to Synchrotron Radiation*. *An Introduction to Synchrotron Radiation* (Wiley, 2019).

[42] Cloetens P (1999). Holotomography: Quantitative phase tomography with micrometer resolution using hard synchrotron radiation x rays. Appl Phys. Lett..

[43] Betz O (2007). Imaging applications of synchrotron X-ray phase-contrast microtomography in biological morphology and biomaterials science. I. General aspects of the technique and its advantages in the analysis of millimetre-sized arthropod structure. J. Microsc..

[44] Cloetens P, Mache R, Schlenker M, Lerbs-Mache S (2006). Quantitative phase tomography of Arabidopsis seeds reveals intercellular void network. Proc. Natl Acad. Sci. USA.

[45] Schindelin J (2012). Fiji: an open-source platform for biological-image analysis. Nat Methods.

[46] Xiong B, Wang B, Xiong S, Lin C, Yuan X (2019). 3D morphological processing for wheat spike phenotypes using computed tomography images. Remote Sens. (Basel).

